# Does Metformin Interfere with Cardiorespiratory and Substrate Oxidation Adaptations to Exercise Training in Metabolic Syndrome Patients? A Randomized Placebo-Controlled Trial

**DOI:** 10.3390/biom16070971

**Published:** 2026-07-01

**Authors:** Jabeur Methnani, Amira Moussa, Wissem Dhahbi, Halil İbrahim Ceylan, Ismail Dergaa, Aymen ElHraiech, Taieb Ach, Imed Latiri, Monia Zaouali, Ali Bouslama, Valentina Stefanica, Asma Omezzine, Ezdine Bouhlel

**Affiliations:** 1 High Institute of Sport and Physical Education of Ksar Said, University of Manouba, Manouba 2010, Tunisia; jabeur.methnani@gmail.com (J.M.); ezdine_sport@yahoo.fr (E.B.); 2 LR12SP11, Biochemistry Department, Sahloul University Hospital, Sahloul 4011, Tunisia; 3 LR19ES09, Laboratory of Exercise Physiology and Physiopathology, from Integrated to Molecular, Biology, Medicine and Health, Faculty of Medicine of Sousse, Sousse 4002, Tunisia; 4 Research Unit (UR22JS01), Sport Sciences, Health and Movement, High Institute of Sport and Physical Education of Kef, University of Jendouba, El Kef 7100, Tunisia; wissem.dhahbi@gmail.com; 5 Training Department, Police College, Qatar Police Academy, Doha 7157, Qatar; 6 Physical Education of Sports Teaching Department, Faculty of Sports Sciences, Atatürk University, Erzurum 25030, Türkiye; 7 Department of Cardiology, Sahloul University Hospital, Sahloul 4011, Tunisia; 8 LR12SP09, Heart Failure Research Laboratory, Farhat Hached University Hospital, Sousse 4000, Tunisia; 9 Department of Endocrinology, University Hospital of Farhat Hached, Sousse 4000, Tunisia; 10 Faculty of Pharmacy of Monastir, University of Monastir, Monastir 5000, Tunisia; 11 Department of Physical Education and Sport, Faculty of Sciences, Physical Education and Informatics, National University of Science and Technology Politehnica Bucharest, Pitesti University Center, 060042 Pitesti, Romania

**Keywords:** metformin, cardiorespiratory fitness, fat oxidation, metabolic syndrome, aerobic exercise training, rating of perceived exertion, biguanides, exercise–drug interaction

## Abstract

Metformin and aerobic exercise are routinely co-prescribed in the management of metabolic syndrome, yet evidence regarding their interaction on cardiorespiratory fitness and substrate oxidation adaptations remains inconsistent. This study aimed to investigate the effects of combined metformin and aerobic training on peak oxygen uptake (VO_2_peak), maximal fat oxidation (MFO), submaximal substrate utilization, and perceived exertion in metformin-naïve adults with metabolic syndrome. In this randomized, placebo-controlled trial, 24 metformin-naïve adults with metabolic syndrome were allocated to receive either metformin (1000 mg/day; MET-EX) or a matched placebo (PLA-EX) combined with supervised aerobic training (5 sessions/week, 60% VO_2_peak, 500 kcal/session) for five weeks; 22 participants (*n* = 11 per group) completed the protocol. VO_2_peak, MFO, fat and carbohydrate oxidation, energy expenditure, and rating of perceived exertion (Borg 6–20) were assessed before and after the intervention. The absolute VO_2_peak gain was modestly attenuated in MET-EX relative to PLA-EX (group × time interaction *p* = 0.042; +0.11 vs. +0.26 L·min^−1^), whereas the interaction for relative VO_2_peak did not reach significance (*p* = 0.088). In contrast, MFO increased substantially more in MET-EX than in PLA-EX (+0.13 vs. +0.04 g·min^−1^; *p* = 0.001), accompanied by greater fat oxidation, energy expenditure, and perceived exertion during moderate-to-high submaximal exercise intensities. Moreover, VO_2_peak improvement was negatively correlated with age exclusively in MET-EX (r = −0.87, *p* < 0.001). These findings suggest that metformin induces a dissociated adaptation profile during aerobic training in metabolic syndrome, characterized by enhanced lipid oxidation alongside attenuated cardiorespiratory adaptations and greater perceived effort, particularly in older individuals.

## 1. Introduction

Metabolic syndrome (MetS) is a cluster of central obesity, insulin resistance, dyslipidemia, and elevated blood pressure that markedly increases the risk of type 2 diabetes mellitus (T2DM) and cardiovascular disease [1]. Beyond its diagnostic components, MetS is characterized by reduced cardiorespiratory fitness [2], mitochondrial dysfunction [3], and impaired metabolic flexibility, including altered lipid oxidation [4,5]. Low VO_2_peak is a strong independent predictor of cardiovascular and all-cause mortality, and diminished capacity to oxidize lipids contributes to ectopic fat accumulation and progressive insulin resistance [6,7]. Accordingly, interventions that improve aerobic capacity and substrate utilization are central to MetS management.

Aerobic exercise training is a cornerstone therapy in MetS and consistently increases VO_2_peak [8], enhances mitochondrial density and oxidative enzyme activity [9], and improves lipid oxidation capacity [10,11]. These adaptations are mediated through repeated metabolic stress and activation of intracellular signaling pathways, including AMP-activated protein kinase (AMPK), which promotes mitochondrial remodeling and improved skeletal muscle oxidative function [12].

Metformin is frequently prescribed in individuals with MetS and dysglycemia due to its ability to reduce hepatic glucose production and improve insulin sensitivity [13]. Its metabolic actions involve inhibition of mitochondrial complex I and indirect AMPK activation [13]. While these effects are beneficial for glycemic control, they may also influence skeletal muscle energetics and substrate utilization [14]. Some studies report increased reliance on fat oxidation during exercise with metformin administration [14], possibly reflecting altered carbohydrate availability and energy sensing [13]. Conversely, other investigations have demonstrated that short-term metformin treatment (up to 6 weeks) may reduce cardiorespiratory fitness in healthy individuals and in metformin-naïve adults with metabolic syndrome [15,16]. However, these findings were not consistent across studies. More recent evidence indicates that prolonged metformin therapy (e.g., 40 weeks) does not significantly alter cardiorespiratory fitness in adults at risk for or living with cardiometabolic disease [17].

In addition, large-scale observational data from the Exercise Testing and Health Outcomes Study (ETHOS) cohort (*n* = 750,302) demonstrated that metformin therapy was associated with a 24% lower mortality risk in individuals with type 2 diabetes, independent of cardiorespiratory fitness (CRF) status [18]. Importantly, mortality risk declined progressively across increasing CRF categories regardless of metformin use, but the reduction was more pronounced when higher fitness levels were combined with metformin therapy, particularly among individuals with lower baseline CRF [18]. These findings suggest that metformin does not impair fitness at the population level and that CRF and metformin exert independent and potentially additive protective effects on long-term outcomes. In individuals with metabolic syndrome and prediabetes, metformin has been shown to attenuate cardiorespiratory fitness improvements to aerobic training [19,20,21]. Similarly, in older adults at risk for type 2 diabetes, 12 weeks of aerobic exercise combined with metformin attenuated the training-induced increase in VO_2_max compared with exercise alone [22]. This effect has been attributed to blunted improvements in skeletal muscle mitochondrial respiration, suggesting interference with exercise-induced mitochondrial remodeling. Supporting this interpretation, experimental animal models and transcriptomic analyses in humans have demonstrated suppression of mitochondrial and angiogenic gene programs when metformin is administered during aerobic training [23], further indicating a potential constraint on oxidative and vascular adaptations [21,23].

However, despite the growing body of literature examining the interaction between metformin and exercise training, important knowledge gaps remain. Previous intervention studies have primarily focused on the effects of combined metformin and exercise training on cardiorespiratory fitness, glycemic control, and insulin sensitivity [24,25,26]. Although some investigations have reported measures of substrate utilization, the effects of metformin on exercise-induced adaptations in maximal fat oxidation, substrate utilization across exercise intensities, and perceived exertion remain incompletely characterized [14]. Moreover, these outcomes have rarely been examined simultaneously within the same intervention, limiting our understanding of whether metformin induces a coherent or dissociated pattern of adaptation during exercise training. Consequently, it remains unclear whether the attenuation of cardiorespiratory fitness improvements reported in some studies is accompanied by alterations in lipid oxidation capacity and perceptual responses to exercise, particularly in metformin-naïve individuals with metabolic syndrome.

Therefore, the present study aimed to investigate the effects of a five-week aerobic training program combined with metformin or placebo on VO_2_peak and lipid oxidation in individuals with metabolic syndrome. We hypothesized that metformin would attenuate exercise-induced improvements in VO_2_peak relative to placebo while concurrently enhancing maximal fat oxidation, thereby producing a dissociated cardiometabolic adaptation profile.

## 2. Materials and Methods

### 2.1. Ethics Approval

This study was reviewed and approved by the Institutional Ethics Committee of the Faculty of Medicine of Sousse (Ref: CEFMS 193/2023, date: 27 July 2023). The study was conducted in accordance with the principles of the Declaration of Helsinki. All participants received detailed information about the study and provided written informed consent before enrolment. The trial was registered in the Pan-African Clinical Trials Registry (PACTR202401506460143, date: 9 January 2024).

### 2.2. Study Population

**Figure 1:**
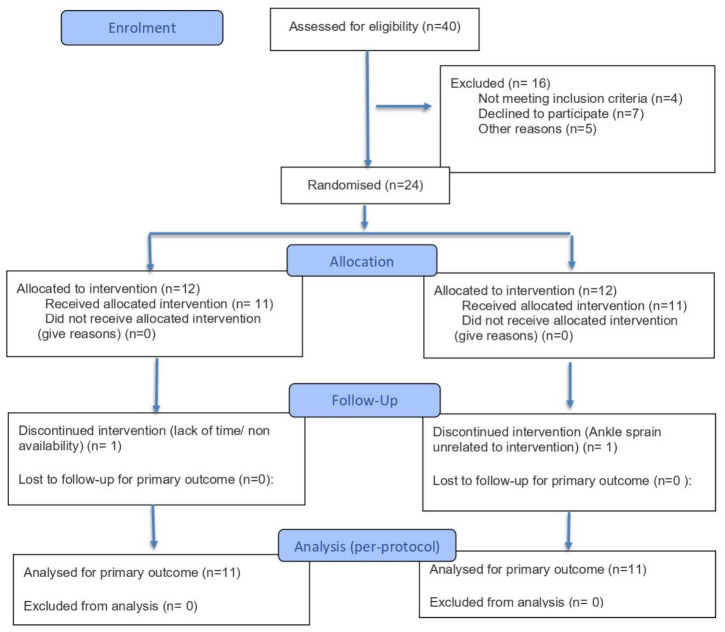
CONSORT flow diagram of participant recruitment, randomization, follow-up, and analysis. Twenty-four metformin-naïve individuals with metabolic syndrome were randomized to receive supervised aerobic exercise combined with metformin (MET-EX, *n* = 12) or placebo (PLA-EX, *n* = 12). Two participants discontinued participation after intervention initiation: one participant in MET-EX withdrew during the first week owing to the recurrence of a pre-existing ankle injury unrelated to the intervention, and one participant in PLA-EX withdrew during the second week because of lack of time and scheduling constraints. Neither participant completed the post-intervention assessments and therefore, both were excluded from the per-protocol analysis. Twenty-two participants (*n* = 11 per group) completed the intervention and were included in the final analyses.

Twenty-four metformin-naïve patients with metabolic syndrome were recruited between March 2023 and March 2024 from primary healthcare centers, the Endocrinology Department of Farhat Hached University Hospital (Sousse), referrals from general practitioners, and social media announcements, and were randomized to one of two intervention groups. Twenty-two participants completed the protocol and were included in the analysis (see flow diagram, Figure 1). No patients, carers, or members of the public were involved in the development of the research question, study design, conduct, outcome selection, data analysis, or dissemination plans for this study.

Metabolic syndrome was defined according to the harmonized criteria [1]. Participants additionally met diagnostic thresholds for prediabetes or type 2 diabetes as defined by the joint International Diabetes Federation/World Health Organization criteria [27], with HbA1c required to be ≤7.5% to ensure inclusion of individuals with controlled, early-stage dysglycemia.

Eligible individuals were physically inactive, defined as not engaging in structured leisure-time exercise and not meeting the World Health Organization recommendation of ≥150 min·week^−1^ of moderate-intensity physical activity [28]—weight stable (<2 kg change in the preceding 3 months), and non-smokers. Individuals with clinically overt or unstable cardiovascular disease, significant renal or hepatic dysfunction, or contraindications to exercise testing or metformin therapy were excluded. All participants were metformin-naïve and were not receiving glucose-lowering pharmacotherapy, insulin therapy, or medications known to influence glucose metabolism. Before enrolment, each participant underwent a medical examination, resting electrocardiogram, and fasting blood chemistry assessment.

### 2.3. Study Design

The data presented in this manuscript were collected as part of a broader experimental investigation examining the interaction between metformin treatment and exercise on glycemic regulation, β-cell function, and insulin sensitivity in adults with metabolic syndrome. The present report focuses specifically on the effects of metformin and time of day on exercise substrate oxidation, maximal fat oxidation (MFO), FATmax, crossover point, energy expenditure, and fasting insulin–glucose-related indices. Other outcomes derived from the same experimental protocol are reported separately. The study was designed as a randomized, placebo-controlled, triple-blind clinical trial. Participants were randomly allocated in a 1:1 ratio to receive one of the two interventions: (1) Exercise + Metformin (MET-EX group) or (2) Exercise + Placebo (PLA-EX group).

#### Randomization

Participants were randomized in a 1:1 ratio using a computer-generated permuted block randomization scheme with variable block sizes of 2, 4, and 6 to ensure balanced group allocation while maintaining allocation unpredictability. The randomization sequence was generated by an independent hospital pharmacist who was not involved in participant recruitment, assessment, exercise supervision, outcome evaluation, or data analysis.

Allocation concealment was maintained through sequentially numbered, sealed opaque envelopes prepared according to the randomization sequence and kept exclusively by the pharmacist. Following participant enrolment, the pharmacist assigned the intervention according to the next envelope in the sequence and dispensed either metformin or placebo in identical prepackaged containers labeled only with the participant identification number. Participants, investigators, exercise supervisors, outcome assessors, and data analysts remained blinded to treatment allocation throughout the study. The allocation code was securely retained by the pharmacist and was not revealed until all data had been collected, and the primary analyses had been completed.

### 2.4. Interventions

The intervention is a combination of two components: a Pharmacological intervention (Metformin [MET] or placebo [PLA] combined with a lifestyle intervention (supervised exercise [EX] resulting in an MET-EX group and a PLA-EX group.

#### 2.4.1. Exercise Training Protocol

A five-week duration was chosen because metformin–exercise interactions on substrate metabolism and perceived exertion are detectable within 28 days [29], enabling capture of early metabolic adaptations within a tightly controlled pharmacological window. This duration is shorter than the 8–12 weeks conventionally required for stable cardiorespiratory adaptation, a constraint explicitly acknowledged in the Limitations. The exercise training program was designed to elicit an energy expenditure of 500 kcal per session at an intensity corresponding to 60% of VO_2_peak, performed five times per week at the Exercise Physiology Laboratory of the Faculty of Medicine of Sousse. Training intensity was prescribed and monitored using an individualized target heart rate corresponding to 60% of VO_2_peak, derived from the linear relationship between heart rate and oxygen uptake established during the baseline maximal graded exercise test for each participant. Heart rate was continuously monitored throughout each session using a Polar V800 monitor (Polar Electro, Kempele, Finland), and exercise intensity was adjusted in real time by the supervising investigator to maintain the participant’s heart rate within ±5 bpm of the prescribed target. Each session began with a standardized 10 min warm-up consisting of joint mobilization exercises, followed by 5 min of stretching. During the first week, training load was progressively increased to familiarize participants with exercise and to reach the prescribed energy expenditure of 500 kcal per session by the end of the week; this target was subsequently maintained until completion of the program. The duration of the main exercise bout, excluding the warm-up, ranged from 45 min to approximately 1 h 40 min, depending on the workload required to attain the prescribed energy expenditure. Adherence was calculated as the percentage of completed sessions over the total prescribed, with ≥80% considered acceptable. All sessions were supervised by the investigators.

#### 2.4.2. Pharmacological Intervention

Metformin was prescribed at a dose of 500 mg twice daily. Participants were instructed to take their dose 30 min before their lunch and dinner based on findings from our group [30] and others [31]. The placebo consisted of 500 mg of starch per tablet, was identical in appearance to the metformin tablets, and was administered according to the same schedule. To preserve blinding, containers were prepared with a variable number of tablets according to the allocation sequence and labeled only with the participant’s identification number. Follow-up visits were scheduled weekly throughout the five-week intervention to verify adherence and dispense subsequent tablet supplies. At each visit, participants were dispensed a variable surplus of tablets exceeding their weekly requirement and were not informed of the exact quantity provided in order to preserve blinding and prevent any inference about the prescribed dosing schedule. Participants were instructed to return all previously dispensed medication containers at each visit. Adherence to the pharmacological intervention was assessed by pill count and was calculated as the proportion of consumed tablets (i.e., dispensed minus returned) relative to the number of tablets that should have been taken according to the prescribed dosing schedule.

### 2.5. Experimental Procedures

Each participant attended three assessment visits before the intervention and two other visits after the intervention that were scheduled at the same time of day. All visits were conducted at the Exercise Physiology Laboratory of the Faculty of Medicine of Sousse, scheduled in the morning between 7:30 and 11 a.m. for each participant to control for diurnal variation in metabolic and cardiorespiratory measurements [32].

#### 2.5.1. Pre-Intervention Assessment

Visit 1 served as the screening and consent visit and included provision of detailed study information, written informed consent, medical history, fasting blood sampling for biochemical screening, anthropometric measurements, and measurement of resting metabolic rate by indirect calorimetry. Participants were instructed to attend Visit 1 in a 12 h fasted state to allow biochemical screening and resting metabolic rate measurement. Visit 2 (≥48 h after Visit 1) included a maximal graded exercise test on a cycle ergometer to determine VO_2_peak and maximal aerobic power (MAP). Visit 3 (24 h after Visit 2) consisted of the submaximal exercise indirect calorimetry test, performed following a 12 h overnight fast.

#### 2.5.2. Post-Intervention Assessment

Following completion of the 5-week training program, participants attended two post-intervention visits that replicated the outcome assessments performed at visits 2 and 3. Visits were scheduled in the same order and at the same time of day as their pre-intervention counterparts to control for diurnal variation. To dissociate chronic training adaptations from any residual acute pharmacological or exercise effects, the timing of post-tests was standardized as follows: the maximal graded exercise test was performed 48 h after the final supervised training session and 48 h after the final metformin (or placebo) dose, and the submaximal exercise indirect calorimetry test was performed 72 h after the final training session and 72 h after the final dose. Participants were instructed to refrain from any structured physical activity, alcohol, and caffeine for 24 h before each test. Compliance with these instructions was verified by self-report.

#### 2.5.3. Dietary Control

A standardized dietary control protocol was applied before all metabolic assessments. 48 h before each test, participants followed an individualized diet matched to their estimated total daily energy expenditure (TDEE), as previously described by our group [30]. Briefly, TDEE was calculated by multiplying each participant’s measured resting metabolic rate by an activity factor of 1.4, corresponding to a sedentary activity level. Macronutrient composition of the prescribed diet was held constant across participants and visits, with 54% of total energy from carbohydrate, 30% from fat, and 16% from protein. Compliance on day 1 was verified by a written 24 h dietary recall submitted electronically to the investigators; compliance on day 2 was confirmed by a supervised video call reviewing intake of the standardized final pre-test meal.

#### 2.5.4. Resting Metabolic Rate

Resting metabolic rate was assessed by indirect calorimetry using standard methodological guidelines [33]. Measurements were performed using the same metabolic system (Metasys TR, Brainware SA, La Valette, France) under controlled resting conditions.

#### 2.5.5. Anthropometric Measurements

Body height was measured to the nearest 1 mm using a wall-mounted stadiometer (SECA 225, Hamburg, Germany) with participants standing upright after a normal expiration. Body mass was assessed using an electronic scale (Polar Electro, Kempele, Finland). Waist circumference was measured using a non-elastic flexible tape at the narrowest point between the lower costal margin and the iliac crest.

#### 2.5.6. Maximal Graded Exercise Test

Participants reported to the laboratory in the morning to perform a graded maximal exercise test. After a 4 min unloaded warm-up, the test was conducted on a pre-calibrated electromagnetically braked cycle ergometer (Excalibur, Lode, Groningen, The Netherlands). The protocol began at 20 W for women and 40 W for men, with increments of 20 W·min^−1^ until volitional exhaustion. Pulmonary gas exchange was measured breath-by-breath using an automated metabolic system (Metasys TR, Brainware SA, La Valette, France). The test was used to determine peak oxygen uptake (VO_2_peak) and Maximal Aerobic Power (MAP). VO_2_peak was defined as the highest value averaged during the last 30 s of the last stage. Heart rate and electrocardiographic activity were continuously monitored throughout the test using a standard 12-lead ECG.

#### 2.5.7. Exercise Indirect Calorimetry

The indirect calorimetry exercise tests were performed in the morning following a 12–13 h overnight fast. Each test began with a 6 min resting measurement followed by five stages of six-minute steady-state cycling at 20, 30, 40, 50, and 60% of MAP determined from the maximal graded exercise test. All tests were performed on the same electromagnetically braked cycle ergometer. Ventilatory and metabolic responses were measured breath-by-breath using the same gas analysis system to ensure methodological consistency.

#### 2.5.8. Indirect Calorimetry Calculations

During exercise indirect calorimetry, VO_2_ and VCO_2_ values from the final 2 min of each workload were averaged and used to calculate non-protein substrate oxidation according to the equations of Péronnet et al. [34]:

- Carbohydrate oxidation: 4.585·VCO_2_ − 3.225·VO_2_
- Fat oxidation: 1.646·VO_2_ − 1.7012·VCO_2_

MFO was defined as the highest absolute rate of fat oxidation obtained across stages. Fatmax was defined as the exercise intensity (%VO_2_peak) at which MFO occurred.

The crossover point was defined as the exercise intensity at which carbohydrate oxidation became the predominant energy source, typically corresponding to ~70% carbohydrate and ~30% fat contribution to total EE, reflected by a respiratory exchange ratio of 0.91 during exercise [35]. This point was estimated by calculating absolute (kcal·min^−1^) and relative (%) contributions of carbohydrate and fat oxidation to total EE using Atwater conversion factors (4 kcal·g^−1^ for carbohydrates and 9 kcal·g^−1^ for fat).

#### 2.5.9. Rating of Perceived Exertion

Perceived exertion was assessed during each stage of the exercise indirect calorimetry test using the Borg 6–20 Rating of Perceived Exertion (RPE) scale, which provides a valid and sensitive measure of subjective exercise intensity across a wide range of workloads [36].

#### 2.5.10. Biochemical Analyses

Blood samples were collected for biochemical analyses using standard procedures. Serum Triglycerides (TG), Total Cholesterol (TC), high-density lipoprotein (HDL)-Cholesterol (C), and insulin were obtained from blood collected in plain tubes after clotting at room temperature. TG and TC were measured using glycerol-3-phosphate oxidase and cholesterol oxidase methods, respectively; HDL-C was assessed by a direct enzymatic colorimetric method, and low-density lipoprotein (LDL)-C was calculated using the Friedewald formula. Plasma glucose was measured from fluoride/oxalate tubes using the hexokinase method. All analytes, except insulin, were measured immediately after centrifugation on an AU680 biochemistry autoanalyzer (Beckman Coulter, Brea, CA, USA). Insulin was determined by electrochemiluminescence immunoassay (Cobas^®^ E411), and HbA1c was measured by nephelometry using the IMMAGE Immunochemistry System (Beckman Coulter, Brea, CA, USA).

#### 2.5.11. Harms

Adverse events were monitored passively throughout the intervention period by recording any spontaneously reported symptoms or events during supervised exercise sessions and study visits. Events were documented by the research team, classified according to type and severity, and reviewed without formal causality adjudication. No systematic harm questionnaire or validated adverse-event scale was used.

### 2.6. Sample Size Calculation

The parent trial was initially designed to evaluate multiple physiological adaptations to combined metformin and exercise training. The current manuscript specifically focuses on cardiorespiratory fitness and substrate oxidation outcomes. Sample size was calculated using Python (statsmodels.stats.power, version 0.14.0). The primary outcome for this article was the pre-to-post intervention change in peak oxygen uptake (VO_2_peak), assessed by paired comparison (pre vs. post) within each treatment arm. Effect size estimation was based on the Exercise + Metformin group reported by Cadeddu [25], as this arm most closely reflects the MET-EX group of the present study (insulin-resistant adults; metformin 1000 mg/day combined with supervised aerobic training). From the study of Cadeddu et al., the pre-to-post change in absolute VO_2_peak in the MEx group was 0.19 L·min^−1^ (95% CI: 0.05–0.33 L·min^−1^; *n* = 21). Because this confidence interval was derived from a paired *t*-test, the standard deviation (SD) of the change scores was back-calculated as SE = (0.33 − 0.05)/[2 × t(0.025, df = 20)] = 0.067 L·min^−1^, then SD = 0.067 × √21 = 0.308 L·min^−1^, yielding Cohen’s d = 0.62. This was adjusted for the paired design using an expected pre–post correlation of r = 0.85, reflecting the high test–retest reliability of VO_2_peak in supervised exercise training protocols. With a two-sided α = 0.05 and 80% target power, a minimum of 9 participants per group (22 total) was required. Anticipating a 30% dropout rate, a recruitment target of 12 per group (24 total) was set. It should be noted that this calculation was based on a within-group paired comparison framework and was not prospectively powered to detect the Group × Time interaction effects that underlie the primary conclusions. With *n* = 11 per group, the study is underpowered for robust detection of interaction effects; the observed interaction η^2^p values (0.191 for VO_2_peak; 0.413 for MFO) are large by conventional benchmarks but should be treated as preliminary estimates pending confirmation in larger trials.

No interim analyses or formal stopping guidelines were prespecified because of the short intervention duration, modest sample size, and fixed recruitment target.

### 2.7. Statistical Analysis

All analyses were performed using IBM SPSS Statistics (Version 23.0, IBM Corp., Armonk, NY, USA), with figures generated in Python (Matplotlib, Seaborn) for visualization purposes only. Statistical significance was set at α = 0.05. Analyses were conducted according to the per-protocol principle and included only participants who completed the intervention and all pre- and post-intervention assessments, so no imputation procedures were considered.

Normality of residuals was assessed using the Shapiro–Wilk test and visual inspection of residual distributions (histograms and Q–Q plots), sphericity was evaluated using Mauchly’s test, and Greenhouse–Geisser corrections were applied to within-participant effects when sphericity was violated. Outliers were screened using studentized residuals (|±3|), and no transformations were applied. Continuous variables are presented as mean ± standard deviation unless otherwise specified. Effect sizes are reported as partial eta-squared (η^2^p) for ANOVA effects and as Pearson’s r for correlations.

MFO, crossover point, Fatmax, and VO_2_peak (in absolute and relative terms) were each analyzed using a 2 (group) × 2 (time) mixed-model ANOVA with group as the between-participant factor and time as the within-participant factor. Main effects of group and time, and the group × time interaction, were evaluated, with η^2^p reported for each effect. When the group × time interaction reached statistical significance, simple-effects contrasts were performed using estimated marginal means with Bonferroni correction to evaluate within-group pre- to post-intervention changes and between-group differences at each time point. Mean differences (or mean changes), 95% confidence intervals, and η^2^p are reported for all significant contrasts.

Fat oxidation, carbohydrate oxidation, energy expenditure, and the percentage contribution of each substrate to total energy expenditure were analyzed using a 2 (group: MET-EX vs. PLA-EX) × 2 (time: pre- vs. post-intervention) × 6 (intensity: rest, 20%, 30%, 40%, 50%, and 60% of maximal aerobic power) mixed-model analysis of variance (ANOVA), with group as the between-participant factor and time and intensity as within-participant factors. The omnibus model evaluated the main effects of group, time, and intensity, as well as the group × time × intensity three-way interaction, with η^2^p reported for each effect. When the three-way interaction reached statistical significance, follow-up contrasts were performed using estimated marginal means with Bonferroni correction: within-group paired comparisons evaluated pre- to post-intervention changes at each exercise intensity, and between-group independent comparisons evaluated MET-EX versus PLA-EX at each time point and intensity. Mean differences, 95% confidence intervals, and η^2^p are reported for all significant contrasts. Partial eta-squared (η^2^p) was interpreted according to Cohen’s scale (small effect: 0.01 < η^2^p, *p* < 0.06, medium effect: 0.06 < η^2^p, *p* < 0.14, and large effect: η^2^p > 0.14).

Pearson product–moment correlations were used to evaluate linear associations between continuous variables, including ΔVO_2_peak with participant age and ΔMFO with ΔVO_2_peak. Correlation coefficients (r) and two-tailed *p*-values are reported separately for each group. Regression lines presented in figures are ordinary least-squares linear fits with shaded 95% confidence bands for the mean predicted response across the range of observed values.

## 3. Results

### 3.1. Participants’ Characteristics

A total of 24 metformin-naïve participants with metabolic syndrome were randomized to MET-EX (*n* = 12) or PLA-EX (*n* = 12) (Figure 1). Of these, 22 participants (*n* = 11 per group) completed the five-week intervention and were included in the per-protocol analysis. Two participants discontinued participation after intervention initiation. One participant in MET-EX withdrew during the first week because of the recurrence of a pre-existing ankle injury unrelated to the intervention, whereas one participant in PLA-EX withdrew during the second week owing to lack of time and scheduling constraints. Neither participant completed the post-intervention assessments; therefore, no post-intervention outcome data were available, and both participants were excluded from the final analysis. Baseline characteristics of the MET-EX and PLA-EX groups are presented in Table 1. The two groups were well matched for age, anthropometric measures, blood pressure, glycemic indices, lipid profile, and baseline cardiorespiratory fitness, with no statistically significant between-group differences for any baseline variable (all *p* > 0.05). Participants were aged 43.09 ± 13.38 years in MET-EX and 46.6 ± 8.5 years in PLA-EX, with a body mass index of 37.9 ± 5.1 kg·m^−2^ and 37.1 ± 4.0 kg·m^−2^, respectively, consistent with class II obesity. Waist circumference (108.0 ± 11.4 vs. 115.4 ± 18.5 cm) confirmed the presence of central adiposity in both groups, and resting blood pressure was elevated in both arms (systolic: 136.5 ± 6.0 vs. 131.8 ± 9.4 mmHg; diastolic: 82.2 ± 7.9 vs. 80.8 ± 6.3 mmHg). Fasting plasma glucose averaged 6.24 ± 0.8 mmol·L^−1^ in MET-EX, and 6.18 ± 0.7 mmol·L^−1^ in PLA-EX, and HbA1c averaged 6.26 ± 0.4% and 6.14 ± 0.2%, respectively, with all participants meeting harmonized criteria for metabolic syndrome and falling within the prediabetes or controlled type 2 diabetes range (HbA1c ≤ 7.5%).

Adherence to the intervention was high in both arms. Compliance with the supervised exercise program—quantified as the percentage of completed training sessions over the five-week protocol—averaged 94.7 ± 1.9% in MET-EX and 95.1 ± 3.1% (95% CI: 93.0–97.2%) in PLA-EX, with no significant between-group difference (*p* > 0.05). Similarly, compliance with the prescribed medication, verified by tablet count at scheduled visits, averaged 90.6 ± 2.4% in MET-EX and 92.6 ± 2.5% in PLA-EX, again with no significant between-group difference (*p* > 0.05). One participant in MET-EX experienced transient nausea on the first day of treatment, which resolved spontaneously by the following day without requiring dose reduction or treatment discontinuation. A separate participant in MET-EX sustained an ankle sprain unrelated to the training program that prevented continuation of the supervised exercise sessions; this participant was withdrawn from the intervention and excluded from the final analysis.

### 3.2. Cardiorespiratory Fitness

**Figure 2:**
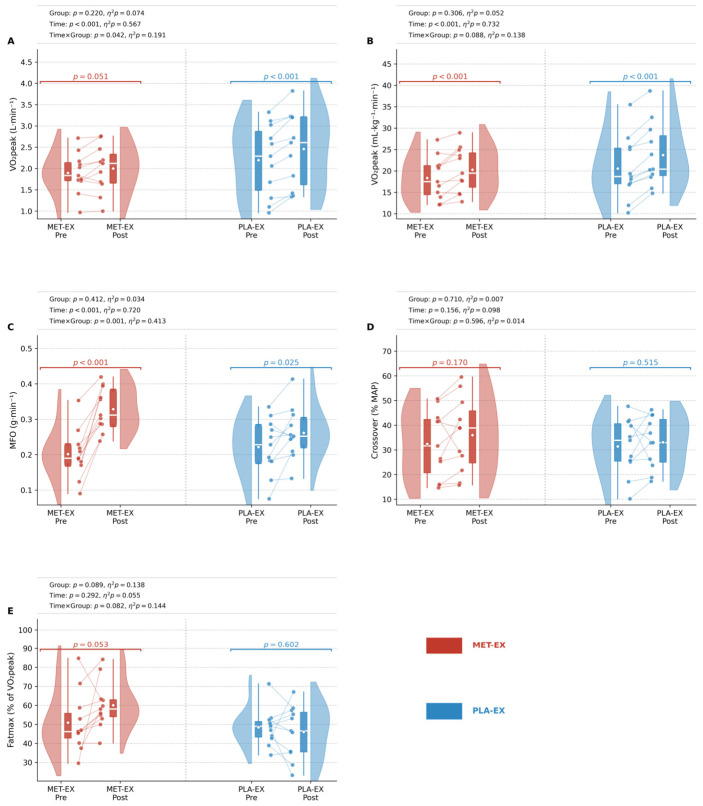
Cardiorespiratory fitness and substrate utilization endpoints before and after the five-week intervention. Raincloud plots are shown for (**A**) absolute peak oxygen uptake ($\dot{V}$O_2_peak, L·min^−1^), (**B**) relative peak oxygen uptake ($\dot{V}$O_2_peak, mL·kg^−1^·min^−1^), (**C**) maximal fat oxidation (MFO, g·min^−1^), (**D**) crossover point expressed as the percentage of maximal aerobic power (% MAP) at which carbohydrate oxidation contributes 70% of total energy expenditure, and (**E**) Fatmax expressed as the percentage of $\dot{V}$O_2_peak at which MFO occurred. Data are shown for the exercise plus metformin group (MET-EX, red) and the exercise plus placebo group (PLA-EX, blue) at pre- and post-intervention. Each panel combines half-violin kernel density estimates, individual participant values connected by paired pre-to-post lines, and slim box plots (median, interquartile range, whiskers to minimum and maximum observed values). The boxed text above each panel reports the main effects of group, time, and the time × group interaction from a 2 (group) × 2 (time) mixed-model ANOVA, with *p* values and partial eta squared (η^2^p). Bracketed *p* values above each condition display within-group pre- vs. post-intervention contrasts from estimated marginal means with Bonferroni correction. Group colours are defined in the legend (bottom right). *n* = 11 per group.

Absolute VO_2_peak (L·min^−1^) showed no significant group effects (Figure 2A; *p* = 0.220, η^2^p = 0.074), a significant effect of time (pre- vs. post-intervention) (*p* < 0.0001, η^2^p = 0.567), and a significant group × time interaction (*p* = 0.042, η^2^p = 0.191).

In MET-EX, absolute VO_2_peak tended to increase from pre- to post-intervention (*p* = 0.051, mean change = 0.106 L·min^−1^, 95% CI: [−0.0004, 0.212], η^2^p = 0.178), whereas in PLA-EX, absolute VO_2_peak increased from pre- to post-intervention (*p* < 0.0001, mean change = 0.26 L·min^−1^, 95% CI: [0.16, 0.37], η^2^p = 0.571). At pre-intervention, absolute VO_2_peak did not differ between MET-EX and PLA-EX (*p* = 0.313), and the same was observed post-intervention (*p* = 0.159).

In relative terms VO_2_peak (mL·kg^−1^·min^−1^) showed no significant group effect (Figure 2B; *p* = 0.306, η^2^p = 0.052), a significant main effect of time (pre- vs. post-intervention) (*p* < 0.0001, η^2^p = 0.732), and a tendency of group × time interaction (*p* = 0.088, η^2^p = 0.138). Although the group × time interaction did not reach statistical significance (*p* = 0.088), there was a trend towards a greater improvement in PLA-EX. Bonferroni-corrected contrasts showed that in MET-EX group, relative VO_2_peak (Figure 2B) changed from pre- to post-intervention (*p* = 0.0008, mean change = 1.9 mL·kg^−1^·min^−1^, 95% CI: [0.9, 2.9], η^2^p = 0.439). In PLA-EX, relative VO_2_peak changed from pre- to post-intervention (*p* < 0.0001, mean change = 3.2 mL·kg^−1^·min^−1^, 95% CI: [2.1, 4.2], η^2^p = 0.678). No pre-intervention (*p* = 0.417, mean difference = −2.2 mL·kg^−1^·min^−1^, 95% CI: [−7.8, 3.4], η^2^p = 0.033), or post-intervention (*p* = 0.225, mean difference = −3.4 mL·kg^−1^·min^−1^, 95% CI: [−9.2, 2.3], η^2^p = 0.073) were found between MET-EX and PLA-EX in relative VO_2_peak (Figure 2B).

### 3.3. MFO

MFO (g·min^−1^) showed no significant between-participant effect of group (Figure 2C; *p* = 0.412, η^2^p = 0.034), a significant main effect of time (pre- vs. post-intervention) (*p* < 0.0001, η^2^p = 0.720), and a significant group × time interaction (*p* = 0.001, η^2^p = 0.413) (Figure 2C).

Bonferroni-corrected contrasts showed that in MET-EX group, MFO changed from pre- to post-intervention (*p* < 0.0001, mean change = 0.127 g·min^−1^, 95% CI: [0.093, 0.161], η^2^p = 0.749). In PLA-EX, MFO changed from pre- to post-intervention (*p* = 0.025, mean change = 0.040 g·min^−1^, 95% CI: [0.006, 0.074], η^2^p = 0.227). At pre-intervention, MFO did not differ between MET-EX and PLA-EX (*p* = 0.536) but at post-intervention, MFO was clearly higher in MET-EX group compared to PLA-EX group (*p* = 0.031, mean difference = 0.067 g·min^−1^, 95% CI: [0.007, 0.127], η^2^p = 0.213).

### 3.4. Crossover Point

The crossover point revealed no significant between-participant effect of group (Figure 2D; *p* = 0.710, η^2^p = 0.007), no significant main effect of time (pre- vs. post-intervention) (*p* = 0.156, η^2^p = 0.098), and no significant group × time interaction (*p* = 0.596, η^2^p = 0.014) (Figure 2D).

### 3.5. Fatmax

Fatmax revealed a marginal between-participant effect of group (Figure 2E; *p* = 0.089, η^2^p = 0.138), no significant main effect of time (pre- vs. post-intervention) (*p* = 0.292, η^2^p = 0.055), and a marginal group × time interaction (*p* = 0.082, η^2^p = 0.144) (Figure 2E).

### 3.6. Fat Oxidation Across Submaximal Exercise Intensities (Rest to 60% MAP)

**Figure 3:**
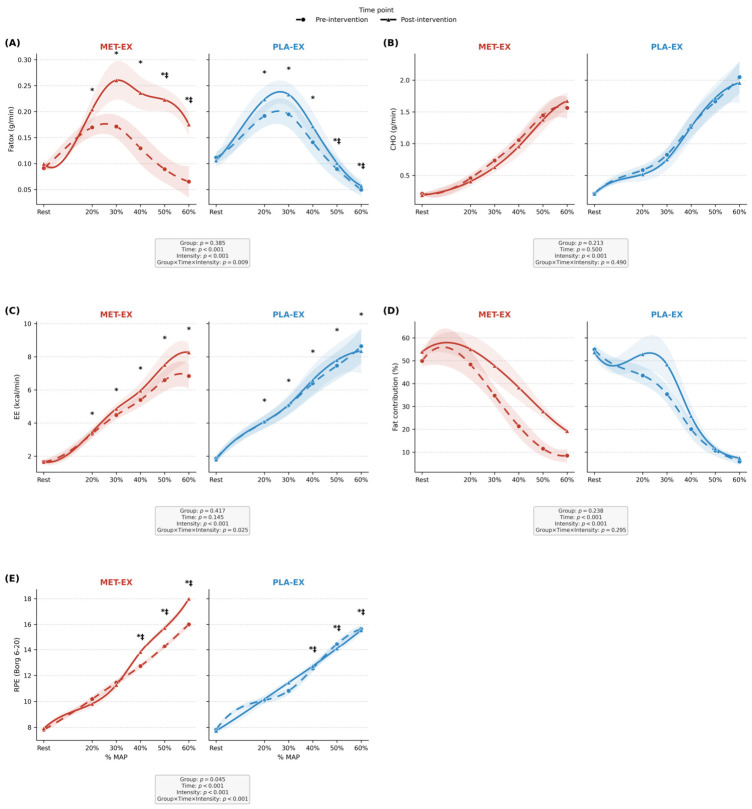
Substrate oxidation, energy expenditure, substrate contribution, and perceived exertion across submaximal exercise intensities before and after the five-week intervention. Panels show (**A**) fat oxidation (g·min^−1^), (**B**) carbohydrate oxidation (CHO, g·min^−1^), (**C**) energy expenditure (EE, kcal·min^−1^), (**D**) percentage contribution of fat to total energy expenditure, and (**E**) rating of perceived exertion (RPE; Borg 6–20 scale), measured at rest and during steady-state cycling at 20%, 30%, 40%, 50%, and 60% of maximal aerobic power (MAP). Within each panel, the exercise plus metformin group (MET-EX, red, left) and the exercise plus placebo group (PLA-EX, blue, right) are shown separately. Dashed lines with open circles denote pre-intervention values; solid lines with triangles denote post-intervention values. Shaded bands represent ±1 SE. Boxed text beneath each parameter reports *p* values for the main effects of group, time, and intensity and the group × time × intensity interaction from a 2 (group) × 2 (time) × 6 (intensity) mixed-model ANOVA. Symbols denote Bonferroni-adjusted post-hoc contrasts and are displayed only when the three-way interaction was statistically significant: * within-group pre- vs. post-intervention (*p* < 0.05); ‡ MET-EX vs. PLA-EX at post-intervention (*p* < 0.05). *n* = 11 per group.

Fat oxidation (g·min^−1^) showed no significant between group effects (Figure 3A *p* = 0.385, η^2^p = 0.038), a significant main effect of time (pre- vs. post-intervention) (*p* < 0.0001, η^2^p = 0.733), a significant main effect of intensity (*p* < 0.0001, η^2^p = 0.584), and a significant group × time × intensity interaction (*p* = 0.009, η^2^p = 0.139).

Follow-up Bonferroni-corrected pairwise contrasts showed that MET-EX raised fat oxidation at 20% MAP (time effect, *p* = 0.027, η^2^p = 0.403, mean change: 0.035 g·min^−1^, 95% CI: [0.005, 0.064]), at 30% MAP (time effect, *p* = 0.012, η^2^p = 0.486, mean change: 0.089 g·min^−1^, 95% CI: [0.025, 0.154]), at 40% MAP (time effect, *p* = 0.013, η^2^p = 0.474, mean change: 0.107 g·min^−1^, 95% CI: [0.027, 0.186]), at 50% MAP (time effect, *p* < 0.0001, η^2^p = 0.929, mean change: 0.134 g·min^−1^, 95% CI: [0.108, 0.160]), and at 60% of MAP (time effect, *p* = 0.0003, η^2^p = 0.737, mean change: 0.110 g·min^−1^, 95% CI: [0.064, 0.157]). In contrast, MET-EX fat oxidation did not change at rest (*p* > 0.05). PLA-EX fat oxidation did not significantly change at any intensity (all *p* > 0.05).

Between-group contrasts (MET-EX vs. PLA-EX) showed that at post-intervention, fat oxidation was higher in MET-EX than in PLA-EX at 50% MAP (between-group, *p* = 0.0003, η^2^p = 0.506), and 60% MAP (between-group, *p* = 0.0003, η^2^p = 0.565) (Figure 3A).

### 3.7. Carbohydrate Oxidation Across Submaximal Exercise Intensities (Rest to 60% MAP)

Carbohydrate oxidation (g·min^−1^) showed no significant between-group effects (Figure 3B; *p* = 0.213, η^2^p = 0.076), no significant main effect of time (pre- vs. post-intervention) (*p* = 0.500, η^2^p = 0.023), a significant main effect of intensity (*p* < 0.0001, η^2^p = 0.800), and no significant group × time × intensity interaction (*p* = 0.490, η^2^p = 0.043).

### 3.8. Energy Expenditure Across Submaximal Exercise Intensities (Rest to 60% MAP)

Mixed ANOVA on energy expenditure (kcal·min^−1^) revealed no significant between group effects (Figure 3C; *p* = 0.417, η^2^p = 0.033), no significant main effect of time (pre- vs. post-intervention) (*p* = 0.145, η^2^p = 0.103), a significant main effect of intensity (*p* <0.0001, η^2^p = 0.808), and a significant group × time × intensity interaction (*p* = 0.025, η^2^p= 0.118).

At post intervention, Bonferroni-corrected pairwise contrasts showed that energy expenditure was higher in MET-EX group at 20% MAP (Figure 3C; *p* =0.041, η^2^p = 0.353, mean change: 0.103 kcal·min^−1^, 95% CI: [0.005, 0.201]), at 30% MAP (time effect, *p* =0.0002, η^2^p = 0.776, mean change: 0.384 kcal·min^−1^, 95% CI: [0.239, 0.529]), at 40% MAP (time effect, *p* = 0.0010, η^2^p = 0.679, mean change: 0.565 kcal·min^−1^, 95% CI: [0.291, 0.838]), at 50% MAP (time effect, *p* < 0.0001, η^2^p = 0.855, mean change: 0.920 kcal·min^−1^, 95% CI: [0.652, 1.187]), and at 60% MAP (time effect, *p* = 0.004, η^2^p = 0.589, mean change: 1.427 kcal·min^−1^, 95% CI: [0.588, 2.267]), but not at rest (*p* > 0.05) compared to pre-intervention. Energy expenditure in the PLA-EX did not change at any intensity (all *p* >0.05).

Between-group contrasts showed no between-group differences for energy expenditure at any intensity at either pre- or post-intervention (Figure 3C; all *p* > 0.10).

### 3.9. Fat Contribution to EE (%) Across Submaximal Exercise Intensities (Rest to 60% MAP)

Mixed ANOVA on fat percentage contribution to energy expenditure revealed no significant between group effects (Figure 3D; *p* = 0.238, η^2^p = 0.069), a significant main effect of time (*p* = 0.0002, η^2^p = 0.510), a significant main effect of intensity (*p* < 0.0001, η^2^p = 0.768), and no significant group × time × intensity interaction (*p* = 0.295, η^2^p = 0.058). The main effect of time was associated with a particularly large effect size (η^2^p = 0.510), indicating a substantial influence of time on the contribution of fat percentage to energy expenditure.

### 3.10. Rating of Perceived Exertion (RPE) Across Submaximal Exercise Intensities (Rest to 60% MAP)

Mixed ANOVA on RPE (Borg 6–20 units) revealed a significant between group effects (Figure 3E; *p* = 0.045, η^2^p = 0.186), a significant main effect of time (pre- vs. post-intervention) (*p* < 0.0001, η^2^p = 0.573), a significant main effect of intensity (*p* < 0.0001, η^2^p = 0.983), and a significant group × time × intensity interaction (*p* < 0.0001, η^2^p = 0.288).

Within-group contrasts showed that RPE was higher in MET-EX group at 40% MAP (time effect, *p* = 0.001, η^2^p = 0.655, mean change: 1.1 Borg units, 95% CI: [0.5, 1.6]), at 50% MAP (time effect, *p* = 0.0002, η^2^p = 0.776, mean change: 1.5 Borg units, 95% CI: [0.9, 2.0]), and at 60% MAP (*p* < 0.0001, η^2^p = 0.917, mean change: 2.0 Borg units, 95% CI: [1.6, 2.4]). In contrast, MET-EX RPE did not change at rest, 20% MAP, 30% MAP (all *p* > 0.05). PLA-EX RPE did not change at any intensity (all *p* > 0.05).

Between-group contrasts showed that at post-intervention, RPE was higher in MET-EX than in PLA-EX at 40% MAP (between-group, *p* = 0.004, η^2^p = 0.389), 50% MAP (between-group, *p* = 0.0006, η^2^p = 0.481), and 60% MAP (between-group, *p* < 0.0001, η^2^p = 0.731).

### 3.11. Correlations

**Figure 4:**
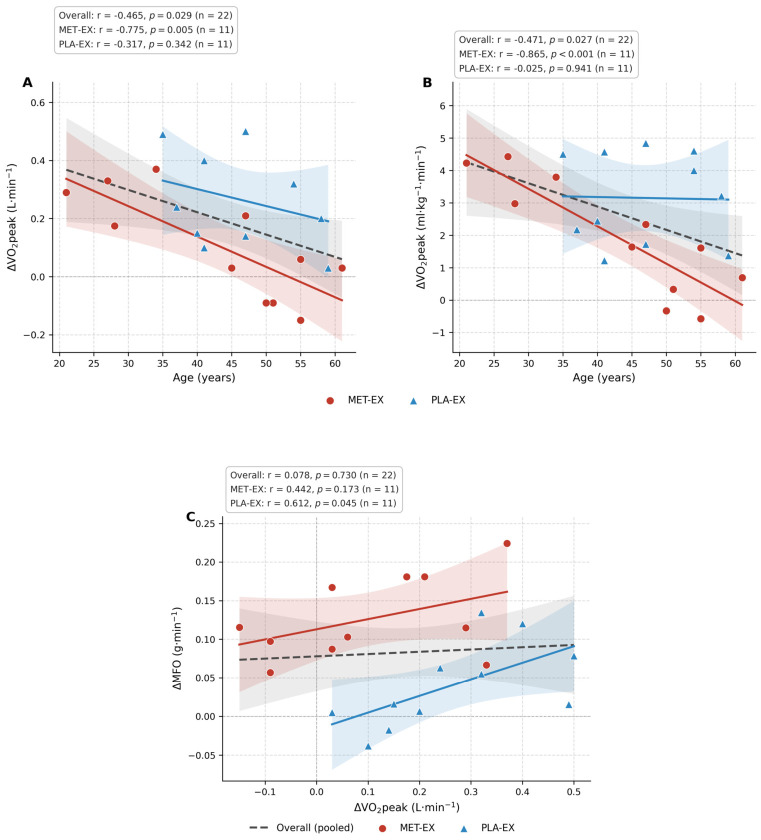
Correlations between training-induced changes in cardiorespiratory fitness, age, and maximal fat oxidation. (**A**) Association between participant age and the absolute change in peak oxygen uptake (ΔVO_2_peak, L·min^−1^). (**B**) Association between participant age and the relative change in peak oxygen uptake (ΔVO_2_peak, mL·kg^−1^·min^−1^). (**C**) Association between the change in absolute peak oxygen uptake (ΔVO_2_peak, L·min^−1^) and the change in maximal fat oxidation (ΔMFO, g·min^−1^). Individual data points are shown for the exercise plus metformin group (MET-EX, red circles) and the exercise plus placebo group (PLA-EX, blue triangles). Solid colored lines represent ordinary least-squares linear regression fits within each group, and the dashed gray line represents the pooled regression across both groups; shaded bands indicate 95% confidence intervals for the mean predicted response. Pearson correlation coefficients (r), two-tailed *p*-values, and sample sizes (*n*) are reported in the inset of each panel for the pooled sample and for each group separately.

The change in VO_2_peak from pre- to post-intervention correlated significantly and negatively with participant age in the MET-EX group, both in absolute terms (r = −0.775, *p* = 0.005; Figure 4A) and in relative terms (r = −0.865, *p* < 0.001; Figure 4B), indicating that older participants in the metformin arm derived smaller cardiorespiratory fitness gains from the training program than younger participants. This age dependence was absent in the PLA-EX group, where neither absolute (r = −0.317, *p* = 0.342) nor relative (r = −0.025, *p* = 0.941) ΔVO_2_peak was significantly associated with age.

The training-induced change in maximal fat oxidation (ΔMFO) was significantly and positively associated with the change in absolute VO_2_peak (ΔVO_2_peak) in the PLA-EX group (r = 0.612, *p* = 0.045; Figure 4C), consistent with the classical coupling between aerobic fitness gains and substrate flexibility improvements. This association did not reach statistical significance in the MET-EX group (r = 0.442, *p* = 0.173), and the within-group regression line was visibly displaced upward relative to PLA-EX, indicating that improvements in MFO occurred in MET-EX independently of, and out of proportion to, gains in VO_2_peak.

To assess whether the correlations were disproportionately influenced by high-leverage observations, a sensitivity analysis was performed using Cook’s distance and Spearman’s rank correlation as a non-parametric robustness check. Cook’s distance values for all participants in MET-EX were below the conventional threshold (4/(n − 2) = 0.44; maximum Di = 0.22), indicating the absence of individually dominant observations. Spearman correlations between age and absolute ΔVO_2_peak in MET-EX were ρ = −0.685 (*p* = 0.020), and between age and relative ΔVO_2_peak were ρ = −0.847 (*p* = 0.001), consistent with the Pearson findings and supporting the robustness of the association to distributional assumptions and individual leverage.

## 4. Discussion

The present study investigated the effects of a five-week supervised aerobic training program combined with metformin or placebo on cardiorespiratory fitness and lipid oxidation in metformin-naïve individuals with metabolic syndrome. The principal findings were, first, aerobic training increased VO_2_peak in both groups, but the improvement was significantly attenuated in MET-EX relative to PLA-EX (group × time interaction *p* = 0.042). Second, this attenuation was strongly age-dependent, with older participants in MET-EX gaining the least fitness (r = −0.865, *p* < 0.001), a relationship absent in PLA-EX. Third, MET-EX produced a substantially greater increase in MFO than PLA-EX (post-intervention MFO was higher in MET-EX, *p* = 0.031). Fourth, RPE increased significantly in MET-EX at 40–60% of peak power output after the intervention but remained unchanged in PLA-EX, with RPE significantly higher in MET-EX at post-intervention.

The observation that absolute VO_2_peak increased significantly in PLA-EX (mean change: +0.26 L·min^−1^, *p* < 0.0001) but only showed a non-significant trend in MET-EX (+0.106 L·min^−1^, *p* = 0.051), alongside a significant group × time interaction (*p* = 0.042, η^2^p = 0.191), is consistent with a growing body of literature documenting metformin interference with exercise-induced aerobic adaptations. This pattern aligns closely with the seminal findings of Konopka et al. [22], who reported that 12 weeks of aerobic exercise training combined with metformin attenuated VO_2_max improvements by approximately 50% in older adults at risk for type 2 diabetes compared with exercise alone. More recently, Moreno-Cabañas et al. [20] demonstrated in individuals with metabolic syndrome and hyperglycemia that chronic metformin treatment was associated with blunted gains in VO_2_max with high-intensity interval training. Extending this evidence to the molecular level, Bruss et al. [23] demonstrated in mice that metformin reduced the number of differentially expressed genes after aerobic exercise training by approximately 50%, attenuating transcriptional programs associated with mitochondrial function, proteostasis, and angiogenesis. A parallel analysis of human resistance exercise data from the MASTERS trial identified BCL6B as a top-ranked exercise-responsive transcription factor suppressed by metformin across both species and exercise modalities, suggesting that metformin may interfere with a conserved transcriptional program linked to vascular and oxidative adaptations to exercise.

The mechanism underlying this functional attenuation, however, remains incompletely characterized and should not be taken as established. Both Konopka et al. [22] and Moreno-Cabañas et al. [20] implicated blunted complex I-linked mitochondrial respiration as the mechanistic driver—an attribution that merits careful scrutiny. Konopka et al. reported that metformin abrogated the exercise-induced increase in maximal complex I-linked skeletal muscle mitochondrial respiration and blunted the improvement in the OXPHOS-to-ETS capacity ratio after 12 weeks of aerobic training, while mitochondrial protein synthesis rates remained unaffected between groups, leading the authors to propose that metformin acts on intrinsic mitochondrial function rather than on biogenesis or abundance. Importantly, however, these findings do not demonstrate direct complex I inhibition. Rather, they indicate an absence of the expected training-induced increase in complex I-linked respiratory capacity. The upstream mechanism responsible for this attenuated adaptation—whether direct complex I interaction, altered cellular redox state, changes in substrate availability, systemic metabolic effects, or other pathways—remains unresolved.

Moreover, the interpretation of human mitochondrial studies requires caution. Recent international efforts to harmonize high-resolution respirometry methodologies have demonstrated that measurements of mitochondrial respiratory capacity are highly sensitive to respiration medium composition, oxygen conditions, sample preparation procedures, and analytical protocols. Indeed, respiratory fluxes may differ substantially despite analysis of samples obtained from the same individual under otherwise standardized conditions. Consequently, quantitative comparison across studies remains challenging, and both positive and negative findings regarding metformin-induced mitochondrial alterations should be interpreted with appropriate caution, particularly in studies with modest sample sizes and substantial interindividual variability [37].

Third, and more directly, Larsen et al. [38] demonstrated that therapeutic doses of metformin (2000 mg/day) did not inhibit complex I respiration ex vivo in permeabilized skeletal muscle fibers from patients with type 2 diabetes, with significant inhibition only emerging at suprapharmacological exposures. Adding further complexity, much of the mechanistic evidence linking metformin to complex I inhibition derives from isolated mitochondria, cultured cells, and hepatic or intestinal tissue models employing metformin exposures substantially exceeding those achieved during routine clinical therapy. Whether concentrations sufficient to meaningfully constrain complex I-linked oxidative phosphorylation are achieved within human skeletal muscle mitochondria during conventional metformin treatment, therefore, remains uncertain.

Nevertheless, recent genetic evidence from Reczek et al. [39] demonstrated that bypassing mitochondrial complex I abolished a substantial proportion of metformin’s acute glucose-lowering effect in vivo, providing compelling evidence that complex I contributes meaningfully to at least some of metformin’s biological actions. However, these experiments were conducted in murine models and primarily addressed glycemic regulation rather than exercise-induced skeletal muscle adaptation. Furthermore, rodents require substantially higher metformin doses than humans to achieve therapeutic effects owing to species-specific pharmacokinetic differences and more rapid drug clearance, complicating direct translation to human exercise physiology. Accordingly, mechanistic inference from preclinical models should be extended to clinical exercise physiology with caution. The metformin dose used in the present study also warrants explicit consideration within this mechanistic and functional context. Participants received 500 mg twice daily (1000 mg/day total), a dose situated at the lower end of the conventional therapeutic range (500–2550 mg/day) and below the doses typically employed in studies reporting attenuation of aerobic exercise adaptations. Konopka et al. [22] used 2000 mg/day (with reduction to 1500 mg/day in participants weighing < 75 kg), Malin et al. [26] employed 2000 mg/day in their combined exercise–metformin protocol in adults with prediabetes, and Moreno-Cabañas et al. [20] studied chronic metformin users with metabolic syndrome whose self-administered dose averaged approximately 1280 mg/day. Critically, Kristensen et al. [40] demonstrated in a randomized crossover study using a substantially higher dose—up to 3000 mg/day—that short-term metformin treatment produced measurable skeletal muscle accumulation reaching approximately 11 µM in exercising muscle, yet did not alter AMPK activity, ACC phosphorylation, phosphocreatine, or glycogen utilization during exercise. While that study did not assess complex I respiration directly, the absence of detectable perturbation in downstream markers of skeletal muscle energy status at a dose three times higher than that used in the present trial further questions whether direct intramuscular energetic disruption through complex I inhibition fully accounts for the physiological effects observed here.

Notably, Cadeddu et al. [25] employed a dose identical to ours (1000 mg/day) in a 12-week supervised aerobic training RCT in insulin-resistant patients and reported that VO_2_peak increased significantly in the metformin group, with no significant between-group difference compared with exercise alone. The convergence between our dose and that of Cadeddu et al. [25]—both yielding less pronounced fitness attenuation than the higher-dose studies—is consistent with a dose-dependent relationship between metformin and its interference with aerobic training adaptation. This dose dependency suggests that the quantitative attenuation observed in the present study may, at least in part, reflect our conservative dosing regimen rather than an inherent pharmacological antagonism of aerobic adaptation, and that at the 1500–2000 mg/day doses conventionally employed in the clinical management of metabolic syndrome and prediabetes, more pronounced modulation of cardiorespiratory and metabolic adaptations may be anticipated. At the same time, our findings confirm that 1000 mg/day was sufficient to induce measurable physiological effects on maximal fat oxidation, substrate utilization, perceived exertion, and VO_2_peak responses—effects that may operate through systemic mechanisms including altered hepatic lactate handling, reduced systemic glucose availability rather than through direct skeletal muscle complex I inhibition, though the present data cannot adjudicate between these pathways. Finally, large-scale observational evidence from the ETHOS cohort (*n* = 750,302) demonstrates that metformin and cardiorespiratory fitness exert independent and additive protective effects on mortality in type 2 diabetes, with progressive mortality benefit across increasing fitness categories regardless of metformin use [18]. These population-level data argue against a clinically meaningful abolition of fitness benefits by metformin and underscore that any modest attenuation of training-induced VO_2_peak gains must be weighed carefully against metformin’s established cardiometabolic and survival benefits.

Notably, our findings regarding cardiorespiratory fitness require nuanced interpretation. In absolute terms, VO_2_peak increased by a mean of +0.106 L·min^−1^ in MET-EX—a change that approached but did not reach statistical significance (*p* = 0.051), compared with a significant increase of +0.26 L·min^−1^ in PLA-EX (*p* < 0.0001), yielding a significant group × time interaction (*p* = 0.042, η^2^p = 0.191). In relative terms, VO_2_peak increased significantly within both groups (MET-EX: +1.9 mL·kg^−1^·min^−1^, *p* = 0.0008; PLA-EX: +3.2 mL·kg^−1^·min^−1^, *p* < 0.0001), though the group × time interaction for relative VO_2_peak did not reach statistical significance (*p* = 0.088), and between-group differences at post-intervention were also non-significant (*p* = 0.159). These statistical nuances are important: the significant interaction for absolute VO_2_peak is driven primarily by the divergence in within-group trajectories rather than by a significant post-intervention between-group difference, and the absence of a significant interaction for relative VO_2_peak further tempers the strength of inference. Given that the study was not prospectively powered to detect group × time interactions, these findings should be considered exploratory and hypothesis-generating, pending confirmation in adequately powered trials.

The more defensible interpretation is therefore not that metformin abolished the fitness response—both groups improved significantly in relative terms—but that it was associated with a quantitative dampening of the aerobic training response: percentage improvements in VO_2_peak were approximately half those observed in PLA-EX in both absolute and relative terms, consistent with attenuation rather than suppression.

This distinction carries direct clinical relevance that extends beyond statistical significance. Epidemiological evidence consistently demonstrates a dose–response relationship between cardiorespiratory fitness and long-term health outcomes. Kodama et al. [41], in a landmark meta-analysis of 33 cohort studies, reported that each 1-MET (3.5 mL·kg^−1^·min^−1^) increment in CRF is associated with a 13–15% reduction in all-cause mortality and cardiovascular disease risk, a finding corroborated and extended by Han et al. [42], who reported an 11–17% reduction in all-cause mortality per 1-MET higher CRF level across 199 cohort studies representing over 20.9 million observations. Although these observational associations should not be interpreted causally, they provide a useful clinical framework for contextualizing the magnitude of the present findings. Within this framework, the improvements in relative VO_2_peak observed in both MET-EX (+1.9 mL·kg^−1^·min^−1^) and PLA-EX (+3.2 mL·kg^−1^·min^−1^) may be considered clinically meaningful, despite the smaller gain observed in the metformin group. Importantly, the between-group difference of 1.3 mL·kg^−1^·min^−1^, while directionally consistent with an attenuation of adaptation, represents a relatively modest absolute difference whose long-term prognostic significance remains uncertain. Accordingly, the present findings should not be interpreted as evidence that metformin negates the clinical benefits of exercise training. This interpretation is reinforced by the ETHOS cohort data [18], which demonstrate that metformin and cardiorespiratory fitness exert independent and additive protective effects on mortality across the full fitness spectrum in type 2 diabetes, with progressive survival benefit regardless of metformin use—arguing against any meaningful abolition of the mortality benefits of improved fitness by metformin. For patients with metabolic syndrome initiating metformin at standard low-to-moderate doses, supervised aerobic training therefore remains a clinically important and effective intervention for improving cardiorespiratory fitness, though optimizing training load, session frequency, and metformin dose to maximize the magnitude of adaptation may be particularly warranted in this population.

The most statistically robust finding of the present study is the group × time interaction for MFO (*p* = 0.001, η^2^p = 0.413), an effect substantially larger than the corresponding interaction observed for absolute VO_2_peak (η^2^p = 0.191). MFO increased by 0.127 g·min^−1^ in MET-EX compared with 0.040 g·min^−1^ in PLA-EX, with a significant between-group difference at post-intervention (*p* = 0.031), indicating that metformin did not merely preserve fat oxidative capacity during training but amplified its adaptation relative to exercise alone.

This finding warrants explicit physiological and clinical contextualization. Impaired fat oxidation and reduced metabolic flexibility are considered hallmark features of metabolic syndrome and have been implicated in the development of ectopic lipid accumulation, insulin resistance, and cardiometabolic dysfunction [4,5]. Consequently, the ability to oxidize fat during exercise—particularly across the submaximal intensity range most relevant to habitual physical activity and exercise prescription—represents an important dimension of metabolic health that is not captured by VO_2_peak alone [4,5,43]. From this perspective, the substantially greater increase in MFO and substrate oxidation rate at submaximal intensities observed in MET-EX suggests that metformin may potentiate specific metabolic adaptations to aerobic training despite its apparent attenuation of cardiorespiratory fitness gains.

Importantly, these findings reinforce the concept of a dissociated adaptation profile rather than a uniform impairment of exercise responsiveness. While improvements in cardiorespiratory fitness appeared quantitatively smaller in MET-EX, adaptations related to fat oxidative metabolism were enhanced. This distinction may be clinically relevant because impaired fat oxidation and reduced metabolic flexibility are recognized features of metabolic syndrome and are closely linked to cardiometabolic dysfunction. Consequently, a greater improvement in MFO may represent a favorable metabolic adaptation that is not captured by changes in VO_2_peak alone.

Nevertheless, caution is warranted when interpreting the clinical significance of this finding. Although enhanced fat oxidative capacity is generally considered indicative of improved metabolic flexibility and oxidative metabolism, no universally accepted prognostic thresholds exist for MFO, and the extent to which the larger MFO response observed in MET-EX translates into superior long-term clinical outcomes remains uncertain. Therefore, the present findings should be viewed primarily as evidence that metformin may differentially modulate aerobic fitness and substrate oxidation adaptations rather than as evidence of superior overall cardiometabolic benefit.

Additional exploratory analyses identified age as a potential modifier of the fitness response to metformin. A negative correlation between age and ΔVO_2_peak was observed in MET-EX in both absolute (r = −0.775, *p* = 0.005) and relative (r = −0.865, *p* < 0.001) terms and was absent in PLA-EX (absolute: r = −0.317, *p* = 0.342; relative: r = −0.025, *p* = 0.941). Given *n* = 11, a sensitivity analysis was performed to assess susceptibility to high-leverage observations (see §3.11). Subject to that analysis, this pattern may reflect greater susceptibility of aged skeletal muscle to metformin-associated bioenergetic constraints [44], but should be treated as hypothesis-generating and requires confirmation in adequately powered studies.

This age dependence is biologically plausible. Aging is accompanied by a progressive decline in skeletal muscle mitochondrial quality and reserve, which may render aged muscle more susceptible to any additional constraint on mitochondrial function [45,46]. In this framework, a comparable degree of pharmacological influence on mitochondrial bioenergetics could be physiologically buffered in younger muscle but become rate-limiting for adaptive remodeling in older muscle, where mitochondrial reserve is already reduced. As mentioned previously, Konopka et al. [22] reported that metformin attenuated mitochondrial respiratory adaptations to aerobic training in older adults (mean age ~62 years), and the MASTERS Trial [47] showed an analogous attenuation of resistance training-induced hypertrophy in older adults. The MET-PREVENT trial similarly reported that metformin neither improved nor, in some domains, attenuated physical performance in older adults with probable sarcopenia [48]. Our findings are broadly consistent with this pattern, although the present study did not examine the molecular mechanisms underlying this response.

The attenuated VO_2_peak response in MET-EX should not be interpreted in isolation from the parallel finding of elevated RPE at moderate-to-high exercise intensities. Beyond direct mitochondrial mechanisms, RPE elevation may itself contribute, indirectly, to the dampened cardiorespiratory adaptation through a behavioral pathway: even within a supervised protocol with maintained session completion, a higher subjective exertion may have led participants in MET-EX to reduce volitional effort during the more demanding portions of training sessions, especially given that exercise intensity was prescribed by individualized heart rate target corresponding to 60% VO_2_peak. Because metformin also elevates submaximal heart rate at any given external workload [29], the heart-rate-based prescription may have further compounded this effect by allowing the target heart rate to be reached at lower mechanical workloads in MET-EX. Over five weeks of repeated training, the convergence of these perceptual and methodological factors may have translated into a reduced cumulative mechanical stimulus delivered to skeletal muscle and thereby contributed to the quantitatively smaller cardiorespiratory adaptation observed in this group. This represents an inherent confounder of heart-rate-based exercise prescription in patients receiving metformin rather than evidence of behavioral non-adherence and should be considered a structural feature of clinical exercise physiology in this population. From a clinical perspective, this finding suggests that patients receiving metformin may experience a greater subjective burden during exercise despite performing comparable external work. Such a dissociation could negatively influence exercise tolerance and potentially compromise long-term adherence, particularly in previously sedentary individuals with metabolic syndrome. Future studies should consider prescribing training at fixed external workloads (e.g., %MAP with verified power output) to dissociate pharmacological from prescription method effects on training adaptation.

This interpretation aligns with prior reports of metformin elevating RPE, blood lactate, and exercise heart rate at submaximal workloads: coordinated responses that signal increased exercise-induced physiological stress. While many of these studies have not documented a measurable reduction in exercise performance, intensity, or workload tolerance accompanying these perceptual and physiological alterations, the crossover trial of Boulé et al. [29] is particularly informative in that it demonstrated after 28 days of metformin (2000 mg/day) in type 2 diabetic patients a similar exercise-induced stress phenotype, characterized by elevated heart rate (+6 bpm), elevated lactate, elevated RPE at all intensities and this was accompanied by a numerical, non-significant reduction in peak and mean torque outputs, suggesting that these perceptual and metabolic alterations were accompanied by a subtle decrement in muscular performance. Consequently, the elevated RPE observed in the present study should not be viewed merely as a perceptual phenomenon but as a potentially clinically relevant response that may influence the effectiveness of exercise prescription when training intensity is regulated using heart rate targets.

The convergence between the elevated RPE response and the substrate oxidation findings extends beyond perceptual and cardiorespiratory outcomes to the broader metabolic phenotype induced by metformin during exercise training. In the present study, MET-EX demonstrated significantly greater fat oxidation across submaximal exercise intensities together with a parallel increase in energy expenditure, despite no detectable change in carbohydrate oxidation. Because fat oxidation yields less ATP per liter of O_2_ than carbohydrate oxidation, the combination of increased lipid utilization and greater oxygen cost at equivalent external workloads is physiologically consistent and suggests that the elevated energy expenditure observed in MET-EX primarily reflects a shift in substrate selection rather than enhanced mechanical work production. This pattern—enhanced fat reliance, increased oxygen cost, and elevated perceived exertion—bears some resemblance to the physiological adaptations reported following ketogenic low-carbohydrate, high-fat dietary interventions, although the underlying mechanisms are likely distinct [49].

While metformin has traditionally been proposed to influence skeletal muscle substrate metabolism through AMPK activation and subsequent facilitation of mitochondrial fatty acid import [50], we consider it unlikely that this mechanism alone fully explains the magnitude of the fat oxidation response observed in the present study. An alternative, though speculative, explanation is that metformin may indirectly alter substrate selection by modifying systemic glucose availability during exercise [13]. Beyond its hepatic effects, metformin substantially increases intestinal glucose uptake and metabolism, such that the intestine has emerged as a major site of metformin action [13,51]. Through the combined effects of reduced hepatic glucose production and increased splanchnic glucose utilization, metformin may create a metabolic environment characterized by lower effective glucose availability, thereby favoring greater reliance on lipid oxidation through substrate competition mechanisms analogous to those described by the Randle cycle. In this framework, the enhanced fat oxidation observed in MET-EX would reflect an adaptive response to altered substrate availability rather than a direct pharmacological stimulation of skeletal muscle fat oxidation pathways.

It should be acknowledged, however, that evidence supporting this hypothesis remains equivocal. Hansen et al. [52], using stable isotope tracer techniques during moderate-intensity exercise, reported that plasma glucose concentrations did not differ between metformin-treated and untreated patients with type 2 diabetes, arguing against a robust metformin-induced reduction in exercise glycaemia. However, these participants had established type 2 diabetes and chronic metformin exposure, whereas the present cohort consisted of metformin-naïve individuals with metabolic syndrome undergoing a five-week combined exercise and metformin intervention. Whether metformin modifies exercise substrate availability differently during the early stages of treatment in this population remains unknown. Because no measurements of blood lactate, glucose kinetics, intestinal glucose utilization, or skeletal muscle metabolism were obtained in the present study, this proposed mechanism should be considered speculative and hypothesis-generating.

Several limitations should be considered when interpreting our findings. The primary statistical limitation is that the prospective power calculation was designed for within-group paired comparisons, not for the Group × Time interaction effects that form the basis of the principal conclusions. With *n* = 11 per group, the study was not adequately powered for reliable detection of interaction effects; the statistical significance of the observed interactions (VO_2_peak: *p* = 0.042, η^2^p = 0.191; MFO: *p* = 0.001, η^2^p = 0.413) should therefore be interpreted with caution, and all findings require confirmation in adequately powered trials.

The five-week intervention duration, while sufficient to detect early adaptations in substrate oxidation and perceived exertion, does not permit definitive attribution of attenuated VO_2_peak gains to pharmacological interference rather than a delayed onset of cardiorespiratory adaptation; trials incorporating multiple assessment time points are required to resolve this ambiguity. The conservative metformin dose (1000 mg/day) sits at the lower end of the therapeutic range, and although this likely contributed to the modest magnitude of cardiorespiratory attenuation, it also precludes characterization of the dose–response relationship between metformin and exercise-induced fitness adaptation.

Another limitation is the inclusion of participants with both prediabetes and early-stage type 2 diabetes. Although all participants met harmonized criteria for metabolic syndrome, were metformin-naïve, and presented relatively mild dysglycemia, differences in underlying metabolic dysfunction between these conditions may have influenced the physiological responses to the intervention. Because the study was not powered for subgroup analyses, potential differences according to glycemic status could not be examined and should be investigated in future adequately powered studies.

Further, dietary adherence was assessed using self-reported dietary records and supervised dietary verification rather than objective biomarkers or direct provision of all meals. Although dietary intake was standardized and compliance was closely monitored, the possibility of reporting bias or unrecorded deviations from the prescribed diet cannot be completely excluded.

Finally, the absence of direct mechanistic readouts, including skeletal muscle mitochondrial respirometry, transcriptomic profiling, and blood lactate kinetics during the calorimetry protocol, limits inference about the molecular basis of the dissociation we describe, and integration of these measurements alongside RPE and substrate utilization should be a priority for subsequent trials.

Several research directions emerge from the present findings that warrant prospective investigation. Methodologically, future trials should integrate skeletal muscle biopsy with high-resolution respirometry and transcriptomic profiling alongside indirect calorimetry, enabling direct linkage between mitochondrial bioenergetics, substrate selection, and perceived effort under metformin. Continuous glucose monitoring during training and tracer-based assessment of exercise glycaemia would clarify whether the elevated perceived effort under metformin reflects a real reduction in systemic glucose availability, an underexamined but mechanistically plausible driver. Designs randomizing patients across metformin dose tiers (500, 1000, 1500, and 2000 mg/day) would establish dose–response relationships that current literature only infers indirectly from across-study comparisons. New target populations deserve attention: lean individuals with type 2 diabetes, in whom the metformin–exercise interaction may differ from the obese metabolic syndrome phenotype; and patients prescribed metformin for emerging non-glycemic indications (polycystic ovary syndrome, gestational diabetes prevention, aging). Finally, the perceptual consequences of metformin should be studied as outcomes in their own right, with ecological assessment of effort during free-living physical activity to test whether elevated RPE meaningfully affects long-term exercise adherence.

## 5. Conclusions

Five weeks of supervised aerobic training combined with metformin in metformin-naïve individuals with metabolic syndrome produced a dissociated cardiometabolic adaptation profile: maximal fat oxidation and fat oxidation across submaximal exercise intensities increased substantially under metformin, whereas the improvement in VO_2_peak appeared attenuated in absolute terms, and the perceptual cost of exercise was amplified, particularly in older participants. The training-induced enhancement of fat oxidation occurred independently of, and out of proportion to, gains in VO_2_peak in MET-EX, indicating an uncoupling between substrate oxidation and cardiorespiratory adaptations under metformin. It should be noted, however, that both groups exhibited significant improvements in relative VO_2_peak, while the evidence for an attenuation of cardiorespiratory fitness was limited to the interaction observed for absolute VO_2_peak. Consequently, the present findings suggest a quantitatively smaller cardiorespiratory adaptation in the metformin group rather than a complete suppression of the training response.

However, because the study was not prospectively powered to detect Group × Time interaction effects, this observation should be considered exploratory and hypothesis-generating. Accordingly, adequately powered randomized controlled trials incorporating mechanistic endpoints are required to confirm the existence, magnitude, clinical significance, and physiological basis of this apparent dissociated adaptation phenotype and to determine the potential influence of metformin dose, age, and metabolic status on exercise training responses.

## Figures and Tables

**Table 1:** Baseline Participant Characteristics.

Variable	MET-EX (*n* = 11)	PLA-EX (*n* = 11)
n (F/M)	11 (7/4)	11 (6/5)
Age (years)	43.09 ± 13.4	46.6 ± 8.5
Body mass (kg)	105.0 ± 19.5	106.8 ± 21.4
Height (m)	1.66 ± 0.08	1.69 ± 0.13
BMI (kg·m^−2^)	37.9 ± 5.1	37.1 ± 4.0
Waist circumference (cm)	108.0 ± 11.4	115.4 ± 18.5
Hip circumference (cm)	116.4 ± 14.5	114.9 ± 7.8
Arterial pressure		
Systolic (mmHg)	136.5 ± 6.0	131.8 ± 9.4
Diastolic (mmHg)	82.2 ± 7.9	80.8 ± 6.3
Cardiorespiratory fitness		
Absolute VO_2_peak (L·min^−1^)	1.9 ± 0.5	2.2 ± 0.8
Relative VO_2_peak (mL·kg^−1^·min^−1^)	18.3 ± 5.0	20.5 ± 7.3
Glycemic indices		
HbA1c (%)	6.26 ± 0.4	6.14 ± 0.2
Fasting glucose (mmol·L^−1^)	6.24 ± 0.8	6.18 ± 0.7
Fasting insulin (pmol·L^−1^)	114.6 ± 18.9	126.4 ± 21.08
Lipid profile		
Total cholesterol (mmol·L^−1^)	5.61 ± 0.85	5.35 ± 0.80
Triglycerides (mmol·L^−1^)	1.53 ± 0.45	1.73 ± 0.50
HDL-cholesterol (mmol·L^−1^)	1.16 ± 0.23	1.14 ± 0.20
LDL-cholesterol (mmol·L^−1^)	3.75 ± 0.78	3.42 ± 0.60

Data are presented as mean ± standard deviation. MET-EX, exercise plus metformin; PLA-EX, exercise plus placebo; BMI, body mass index; VO_2_peak, peak oxygen uptake; HbA1c, glycated hemoglobin; HDL, high-density lipoprotein; LDL, low-density lipoprotein. No statistically significant differences were observed between the MET-EX and PLA-EX groups at baseline for any participant characteristic (all *p* > 0.05).

## Data Availability

The data supporting the findings of this study will be made available from the corresponding author upon reasonable request.

## Abbreviations

The following abbreviations are used in this manuscript:

VO_2_peak	Peak oxygen uptake
MFO	Maximal fat oxidation
RPE	Rating of perceived exertion
MET-EX	Exercise plus metformin (group)
PLA-EX	Exercise plus placebo (group)
MetS	Metabolic syndrome
T2DM	Type 2 diabetes mellitus
AMPK	AMP-activated protein kinase
CRF	Cardiorespiratory fitness
MET	Metformin
PLA	Placebo
EX	Exercise
MAP	Maximal aerobic power
PPO	Peak power output
VO_2_	Oxygen uptake
VCO_2_	Carbon dioxide production
Fatmax	Exercise intensity at which MFO occurs
COP	Crossover point
EE	Energy expenditure
CHO	Carbohydrate oxidation
TDEE	Total daily energy expenditure
RMR	Resting metabolic rate
ECG	Electrocardiogram
TG	Triglycerides
TC	Total cholesterol
HDL-C	High-density lipoprotein cholesterol
LDL-C	Low-density lipoprotein cholesterol
HbA1c	Glycated hemoglobin
BMI	Body mass index
ΔVO_2_peak	Change in peak oxygen uptake
ΔMFO	Change in maximal fat oxidation
ATP	Adenosine triphosphate
BCL6B	B-cell lymphoma 6 member B
PACTR	Pan-African Clinical Trials Registry
CEFMS	Comité d’Éthique de la Faculté de Médecine de Sousse
